# 1-Benzoyl-2-thio­biuret

**DOI:** 10.1107/S1600536812000621

**Published:** 2012-01-14

**Authors:** Sung Kwon Kang, Nam Sook Cho, Min Kyeong Jeon

**Affiliations:** a Department of Chemistry, Chungnam National University, Daejeon 305-764, Republic of Korea

## Abstract

In the title compound [systematic name: *N*-(carbamoyl­carb­a­mo­thio­yl)benzamide], C_9_H_9_N_3_O_2_S, the benzoyl and terminal urea fragments adopt *cisoid* and *transoid* conformations, respectively, with respect to the S atom. The benzoyl and thio­biuret groups are almost coplanar, making a dihedral angle of 8.48 (5)°. The mol­ecular structure is stabilized by an intra­molecular N—H⋯O hydrogen bond. In the crystal, N—H⋯O and N—H⋯S hydrogen bonds link the mol­ecules into a sheet parallel to the *bc* plane.

## Related literature

For structures and reactivity of thia­diazole derivatives, see: Cho *et al.* (1991*a* ▶,*b* ▶, 1996 ▶); Parkanyi *et al.* (1989 ▶). For the biological activity of thia­diazole derivatives, see: Piskala *et al.* (2004 ▶); Castro *et al.* (2008 ▶).
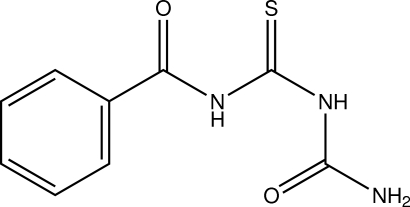


## Experimental

### 

#### Crystal data

C_9_H_9_N_3_O_2_S
*M* *_r_* = 223.25
Monoclinic, 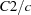

*a* = 10.4583 (3) Å
*b* = 12.8103 (4) Å
*c* = 16.1830 (5) Å
β = 106.693 (1)°
*V* = 2076.73 (11) Å^3^
*Z* = 8
Mo *K*α radiation
μ = 0.30 mm^−1^
*T* = 296 K
0.29 × 0.24 × 0.21 mm

#### Data collection

Bruker SMART CCD area-detector diffractometer
Absorption correction: multi-scan (*SADABS*; Bruker, 2002 ▶) *T*_min_ = 0.911, *T*_max_ = 0.934
7908 measured reflections
1866 independent reflections
1369 reflections with *I* > 2σ(*I*)
*R*_int_ = 0.037

#### Refinement

*R*[*F* ^2^ > 2σ(*F* ^2^)] = 0.039
*wR*(*F* ^2^) = 0.114
*S* = 1.09
1866 reflections
152 parameters
H atoms treated by a mixture of independent and constrained refinement
Δρ_max_ = 0.39 e Å^−3^
Δρ_min_ = −0.48 e Å^−3^

### 

Data collection: *SMART* (Bruker, 2002 ▶); cell refinement: *SAINT* (Bruker, 2002 ▶); data reduction: *SAINT*; program(s) used to solve structure: *SHELXS97* (Sheldrick, 2008 ▶); program(s) used to refine structure: *SHELXL97* (Sheldrick, 2008 ▶); molecular graphics: *ORTEP-3* (Farrugia, 1997 ▶); software used to prepare material for publication: *WinGX* (Farrugia, 1999 ▶).

## Supplementary Material

Crystal structure: contains datablock(s) global, I. DOI: 10.1107/S1600536812000621/is5045sup1.cif

Structure factors: contains datablock(s) I. DOI: 10.1107/S1600536812000621/is5045Isup2.hkl

Supplementary material file. DOI: 10.1107/S1600536812000621/is5045Isup3.cml

Additional supplementary materials:  crystallographic information; 3D view; checkCIF report

## Figures and Tables

**Table 1:** Hydrogen-bond geometry (Å, °)

*D*—H⋯*A*	*D*—H	H⋯*A*	*D*⋯*A*	*D*—H⋯*A*
N9—H9⋯O14	0.89 (2)	1.82 (2)	2.617 (2)	147 (2)
N12—H12⋯O8^i^	0.88 (2)	2.11 (2)	2.946 (2)	158.7 (18)
N15—H15*A*⋯O8^i^	0.91 (3)	2.22 (3)	3.025 (2)	147 (2)
N15—H15*A*⋯S11^i^	0.91 (3)	2.61 (3)	3.312 (2)	134 (2)
N15—H15*B*⋯O14^ii^	0.87 (3)	2.08 (3)	2.943 (2)	177 (2)

Symmetry codes: (i) 
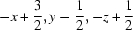
; (ii) 
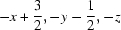
.

**Fig. 1.**
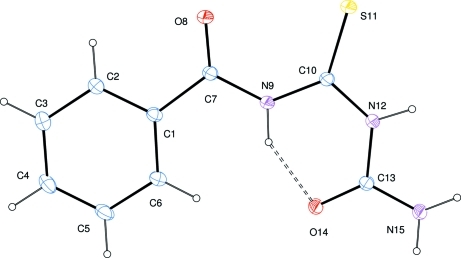
Molecular structure of the title compound, showing the atom-numbering scheme and 30% probability ellipsoids. Intramolecular N—H···O hydrogen bond is indicated by a dashed line.

**Fig. 2.**
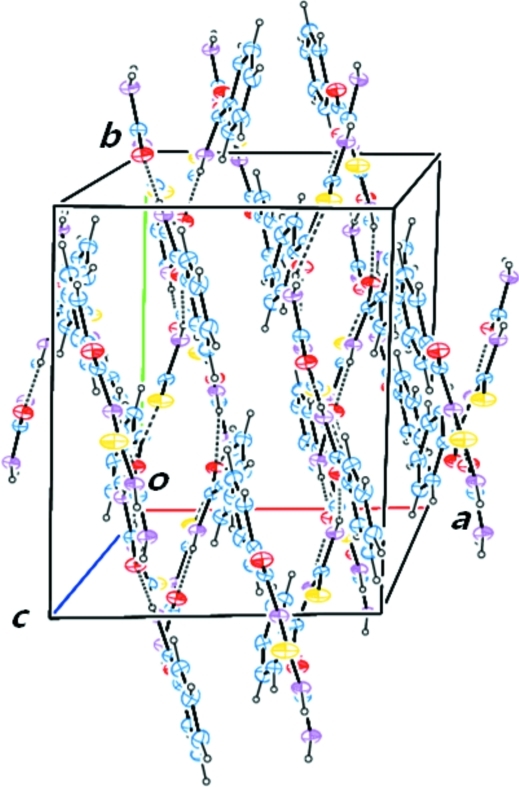
Part of the packing diagram of the title compound, showing a molecular sheet formed by intermolecular N—H···O and N—H···S hydrogen bonds (dashed lines).

## supplementary crystallographic information

### Comment

On the basis of the well known analogy between a –CH=CH– group in benzenoid hydrocarbon and the bivalent sulfur –S–, in their heterocyclic sulfur-containing counterpart (e.g, thiophene is the isoelectronic analog of benzene), 5-amino-2*H*-1,2,4-thiadiazol-3-one is the analog of cytosine. As an analog of cytosine, the tautomeric structure and reactivity of this compound have been examined (Cho *et al.*, 1991*a*,*b*, 1996). Within the framework of our interest in the synthesis of novel potential antimetablites of nucleic acid components which would possess cytostatic and/or antiviral activity, we have synthesized acylonuclesides (Parkanyi *et al.*, 1989). Derivatives of 5-amino-2*H*-1,2,4-thiadiazol-3-one have recently arrested the attention on the antibacterial activity, potential carcinogenicity, and kinase inhibitor activity (Piskala *et al.*, 2004; Castro *et al.*, 2008). The title compound, 1-benzoyl-2-thiobiuret (I), is an intermediate for the formation of the thiobiuret which is a starting material to produce 5-amino-2*H*-1,2,4-thiadizolin-3-one *via* oxidative ring closure reaction.

The dihedral angle between the benzoyl unit (C1–C7/O8 atoms) and thiobiuret group (N9–N15 atoms) is 8.48 (5)°. The carbonyl-O8 and S11 atoms are positioned *syn* to each other, however, carbonyl-O14 atom is *anti* to S11 atom (Fig. 1). The intramolecular N9—H9···O14 hydrogen bond stabilizes the molecule (Fig. 1 and Table 1). The intermolecular N—H···O and N—H···S hydrogen bonds link the molecules into a sheet parallel to the *bc* plane (Fig. 2 and Table 1). The carbonyl-O atoms accept two hydrogen bonds from –NH groups.

### Experimental

To warm solution of potassium thiocyanate (48.0 g, 0.49 mole) in acetone (400 ml), benzoyl chloride (48 mL, 58.2 g, 0.41 mole) was added dropwise. Immediately upon the addition of benzoyl chloride, the solution became milky white and milky yellow when the addition had been completed. The mixture was stirred for 3.5 h at 50 °C and it was left to cool to room temperature. The precipitated potassium chloride was filtered off with suction. The amber filtrate was heated to 55 °C for 5 h with urea (24.0 g, 0.40 mole), the resulting solution was cooled to room temperature and then placed in an ice bath for several hours. The solution was stirred periodically and the walls of the flask were scratched to induce crystallization. The cold mixture was filtered to give 1-benzoyl-2-thiobiuret (27.0 g, 30% yield) as a bright yellow solid. Recrystallization from acetonitrile-methanol (10:1) afforded the yellow crystals suitable for X-ray diffraction, mp 174–175 °C, ^1^H NMR (DMSO-d_6_, p.p.m.): 3.7 (s, 4H, NH_2_ + 2NH), 8.1–8.5 (m, 5H, Ph).

### Refinement

H atoms of the NH and NH_2_ groups were located in a difference Fourier map and refined freely [refined distances = 0.87 (3)–0.91 (3) Å]. Other H atoms were positioned geometrically and refined using a riding model, with C—H = 0.93 Å, and with *U*_iso_(H) = 1.2*U*_eq_(C).

### Crystal data

**Table:**

C_9_H_9_N_3_O_2_S	*F*(000) = 928
*M**_r_* = 223.25	*D*_x_ = 1.428 Mg m^−^^3^
Monoclinic, *C*2/*c*	Mo *K*α radiation, λ = 0.71073 Å
Hall symbol: -C 2yc	Cell parameters from 2609 reflections
*a* = 10.4583 (3) Å	θ = 3.2–28.5°
*b* = 12.8103 (4) Å	µ = 0.30 mm^−^^1^
*c* = 16.1830 (5) Å	*T* = 296 K
β = 106.693 (1)°	Block, yellow
*V* = 2076.73 (11) Å^3^	0.29 × 0.24 × 0.21 mm
*Z* = 8	

### Data collection

**Table:**

Bruker SMART CCD area-detector diffractometer	1369 reflections with *I* > 2σ(*I*)
graphite	*R*_int_ = 0.037
φ and ω scans	θ_max_ = 25.5°, θ_min_ = 2.6°
Absorption correction: multi-scan (*SADABS*; Bruker, 2002)	*h* = −12→8
*T*_min_ = 0.911, *T*_max_ = 0.934	*k* = −13→15
7908 measured reflections	*l* = −19→10
1866 independent reflections	

### Refinement

**Table:**

Refinement on *F*^2^	Primary atom site location: structure-invariant direct methods
Least-squares matrix: full	Secondary atom site location: difference Fourier map
*R*[*F*^2^ > 2σ(*F*^2^)] = 0.039	Hydrogen site location: inferred from neighbouring sites
*wR*(*F*^2^) = 0.114	H atoms treated by a mixture of independent and constrained refinement
*S* = 1.09	*w* = 1/[σ^2^(*F*_o_^2^) + (0.0586*P*)^2^ + 0.3051*P*] where *P* = (*F*_o_^2^ + 2*F*_c_^2^)/3
1866 reflections	(Δ/σ)_max_ < 0.001
152 parameters	Δρ_max_ = 0.39 e Å^−^^3^
0 restraints	Δρ_min_ = −0.48 e Å^−^^3^

### Special details

**Table:**

Geometry. All e.s.d.'s (except the e.s.d. in the dihedral angle between two l.s. planes) are estimated using the full covariance matrix. The cell e.s.d.'s are taken into account individually in the estimation of e.s.d.'s in distances, angles and torsion angles; correlations between e.s.d.'s in cell parameters are only used when they are defined by crystal symmetry. An approximate (isotropic) treatment of cell e.s.d.'s is used for estimating e.s.d.'s involving l.s. planes.
Refinement. Refinement of *F*^2^ against ALL reflections. The weighted *R*-factor *wR* and goodness of fit *S* are based on *F*^2^, conventional *R*-factors *R* are based on *F*, with *F* set to zero for negative *F*^2^. The threshold expression of *F*^2^ > σ(*F*^2^) is used only for calculating *R*-factors(gt) *etc*. and is not relevant to the choice of reflections for refinement. *R*-factors based on *F*^2^ are statistically about twice as large as those based on *F*, and *R*- factors based on ALL data will be even larger.

### Fractional atomic coordinates and isotropic or equivalent isotropic displacement parameters (Å^2^)

**Table:**

	*x*	*y*	*z*	*U*_iso_*/*U*_eq_	
C1	0.61686 (19)	0.18189 (16)	0.04253 (11)	0.0448 (5)	
C2	0.5827 (2)	0.28638 (18)	0.03339 (13)	0.0552 (6)	
H2	0.5944	0.3279	0.0822	0.066*	
C3	0.5314 (2)	0.3297 (2)	−0.04751 (14)	0.0660 (7)	
H3	0.5089	0.4001	−0.053	0.079*	
C4	0.5136 (2)	0.2690 (2)	−0.11972 (13)	0.0667 (7)	
H4	0.4788	0.2983	−0.1742	0.08*	
C5	0.5469 (2)	0.1655 (2)	−0.11173 (13)	0.0674 (7)	
H5	0.5345	0.1247	−0.1609	0.081*	
C6	0.5988 (2)	0.12116 (19)	−0.03144 (12)	0.0587 (6)	
H6	0.6217	0.0508	−0.0266	0.07*	
C7	0.6705 (2)	0.14132 (16)	0.13236 (11)	0.0470 (5)	
O8	0.67319 (17)	0.19421 (11)	0.19501 (8)	0.0635 (5)	
N9	0.71681 (18)	0.03965 (14)	0.13835 (10)	0.0505 (5)	
H9	0.718 (2)	0.0044 (17)	0.0909 (16)	0.069 (7)*	
C10	0.7608 (2)	−0.02204 (16)	0.21000 (12)	0.0483 (5)	
S11	0.77062 (8)	0.00861 (5)	0.31016 (3)	0.0797 (3)	
N12	0.79787 (18)	−0.12067 (14)	0.19313 (10)	0.0521 (5)	
H12	0.8182 (19)	−0.1644 (17)	0.2370 (13)	0.048 (6)*	
C13	0.7888 (2)	−0.16766 (17)	0.11369 (12)	0.0487 (5)	
O14	0.75791 (17)	−0.11737 (11)	0.04566 (8)	0.0623 (5)	
N15	0.8183 (2)	−0.26820 (15)	0.11896 (13)	0.0567 (5)	
H15A	0.821 (2)	−0.307 (2)	0.1667 (17)	0.079 (8)*	
H15B	0.799 (2)	−0.3006 (19)	0.0700 (16)	0.073 (7)*	

### Atomic displacement parameters (Å^2^)

**Table:**

	*U*^11^	*U*^22^	*U*^33^	*U*^12^	*U*^13^	*U*^23^
C1	0.0499 (12)	0.0499 (13)	0.0359 (9)	−0.0032 (9)	0.0143 (8)	−0.0002 (8)
C2	0.0676 (14)	0.0537 (14)	0.0420 (10)	0.0019 (11)	0.0121 (10)	0.0022 (9)
C3	0.0755 (16)	0.0629 (16)	0.0553 (13)	0.0088 (12)	0.0120 (11)	0.0133 (11)
C4	0.0681 (15)	0.089 (2)	0.0405 (11)	0.0116 (14)	0.0108 (10)	0.0141 (11)
C5	0.0719 (15)	0.091 (2)	0.0353 (10)	0.0112 (14)	0.0090 (10)	−0.0051 (11)
C6	0.0728 (14)	0.0609 (14)	0.0390 (10)	0.0069 (11)	0.0106 (10)	−0.0048 (9)
C7	0.0595 (12)	0.0436 (12)	0.0370 (10)	−0.0049 (9)	0.0123 (9)	−0.0008 (8)
O8	0.1056 (13)	0.0474 (9)	0.0352 (7)	0.0069 (8)	0.0168 (7)	−0.0053 (6)
N9	0.0797 (13)	0.0404 (11)	0.0314 (8)	−0.0001 (9)	0.0160 (8)	−0.0016 (7)
C10	0.0670 (13)	0.0382 (12)	0.0379 (10)	−0.0111 (9)	0.0122 (9)	−0.0014 (8)
S11	0.1564 (8)	0.0453 (4)	0.0330 (3)	0.0008 (4)	0.0199 (3)	−0.0030 (2)
N12	0.0813 (13)	0.0401 (10)	0.0338 (8)	−0.0013 (9)	0.0148 (8)	0.0025 (7)
C13	0.0654 (13)	0.0445 (13)	0.0395 (10)	0.0004 (10)	0.0206 (9)	−0.0001 (8)
O14	0.1069 (13)	0.0474 (9)	0.0381 (7)	0.0104 (8)	0.0296 (7)	0.0046 (6)
N15	0.0886 (14)	0.0447 (12)	0.0414 (10)	0.0072 (10)	0.0262 (9)	0.0034 (8)

### Geometric parameters (Å, °)

**Table:**

C1—C2	1.382 (3)	C7—O8	1.213 (2)
C1—C6	1.394 (3)	C7—N9	1.383 (3)
C1—C7	1.493 (2)	N9—C10	1.369 (2)
C2—C3	1.381 (3)	N9—H9	0.89 (2)
C2—H2	0.93	C10—N12	1.372 (3)
C3—C4	1.371 (3)	C10—S11	1.642 (2)
C3—H3	0.93	N12—C13	1.398 (2)
C4—C5	1.368 (3)	N12—H12	0.88 (2)
C4—H4	0.93	C13—O14	1.236 (2)
C5—C6	1.379 (3)	C13—N15	1.321 (3)
C5—H5	0.93	N15—H15A	0.91 (3)
C6—H6	0.93	N15—H15B	0.87 (3)
			
C2—C1—C6	118.77 (18)	O8—C7—N9	122.94 (17)
C2—C1—C7	117.03 (17)	O8—C7—C1	122.10 (19)
C6—C1—C7	124.20 (19)	N9—C7—C1	114.96 (16)
C3—C2—C1	120.6 (2)	C10—N9—C7	128.94 (17)
C3—C2—H2	119.7	C10—N9—H9	110.5 (15)
C1—C2—H2	119.7	C7—N9—H9	120.5 (14)
C4—C3—C2	120.1 (2)	N9—C10—N12	114.10 (17)
C4—C3—H3	120	N9—C10—S11	127.48 (17)
C2—C3—H3	120	N12—C10—S11	118.40 (15)
C5—C4—C3	120.0 (2)	C10—N12—C13	129.15 (17)
C5—C4—H4	120	C10—N12—H12	116.3 (13)
C3—C4—H4	120	C13—N12—H12	113.8 (13)
C4—C5—C6	120.6 (2)	O14—C13—N15	124.21 (19)
C4—C5—H5	119.7	O14—C13—N12	121.74 (19)
C6—C5—H5	119.7	N15—C13—N12	114.05 (18)
C5—C6—C1	119.9 (2)	C13—N15—H15A	122.3 (16)
C5—C6—H6	120	C13—N15—H15B	114.8 (16)
C1—C6—H6	120	H15A—N15—H15B	117 (2)

### Hydrogen-bond geometry (Å, °)

**Table:**

*D*—H···*A*	*D*—H	H···*A*	*D*···*A*	*D*—H···*A*
N9—H9···O14	0.89 (2)	1.82 (2)	2.617 (2)	147 (2)
N12—H12···O8^i^	0.88 (2)	2.11 (2)	2.946 (2)	158.7 (18)
N15—H15A···O8^i^	0.91 (3)	2.22 (3)	3.025 (2)	147 (2)
N15—H15A···S11^i^	0.91 (3)	2.61 (3)	3.312 (2)	134 (2)
N15—H15B···O14^ii^	0.87 (3)	2.08 (3)	2.943 (2)	177 (2)

Symmetry codes: (i) −*x*+3/2, *y*−1/2, −*z*+1/2; (ii) −*x*+3/2, −*y*−1/2, −*z*.
